# Spatial distribution of the four invasive plants and their impact on natural communities’ dynamics across the arid and semi-arid environments in northwest Pakistan

**DOI:** 10.3389/fpls.2023.1207222

**Published:** 2023-08-25

**Authors:** Nasrullah Khan, Rafi Ullah, Mohammad K. Okla, Mostafa A. Abdel-Maksoud, Ibrahim A. Saleh, Hashem A. Abu-Harirah, Tareq Nayef AlRamadneh, Hamada AbdElgawad

**Affiliations:** ^1^ Department of Botany, University of Malakand, Chakdara, Khyber Pakhtunkhwa, Pakistan; ^2^ University of Malakand, Chakdara, Pakistan; ^3^ Department of Botany and Microbiology, College of Science, King Saud University, Riyadh, Saudi Arabia; ^4^ Faculty of Science, Zarqa University, Zarqa, Jordan; ^5^ Department of Medical Laboratory Sciences, Faculty of Allied Medical Sciences, Zarqa University, Zarqa, Jordan; ^6^ Laboratory for Integrated Molecular Plant Physiology Research, Department of Biology, Faculty of Sciences, University of Antwerp, Antwerp, Belgium

**Keywords:** non-native species, floristic diversity, environmental variables, successful invaders, arid and semi-arid environments

## Abstract

**Introduction:**

Non-native species are globally successful invaders with negative impacts on vegetation communities’ social, economic, and ecological values. Hence, the current research was carried out to assess the spatial distribution patterns and vegetative diversity of the four non-native species in severely invaded areas of the semi-arid parts of northern Pakistan.

**Methods:**

The research was conducted using data from 1065 plots spread across 165 sites. These sites represented habitats throughout Northern Province, such as farm countryside, highlands, and abandoned places in rural and urban areas.

**Results and discussion:**

The communities were floristically diverse, represented by 107 plant species, and dominated mainly by annual and perennial life forms with herbaceous habits. Similarly, the floristic structure shows significant variation tested by the χ2 test (P< 0.05) for plant status, life forms, life cycle, and habitat base distribution. In addition, the diversity indices show significant variation having the highest diversity in C-III (*P. hysterophorus*-dominated sites) and lowest in C-IV (*S. marianum*-dominated sites, i.e., primarily pure communities), indicating non-native species may increase or decrease site diversity. The diversity communities were further supported by higher quantities of soil nutrients, i.e., organic percentage (2.22 ± 0.04). Altitude, soil nutrients, and texture were shown to be the environmental factors most associated with communities that non-native species had invaded.

**Recommendation:**

It is recommended that relevant, additional soil and climatic parameters be integrated into species distribution models to improve our understanding of the ecological niches of different species and to make a collective approach for preserving and conserving native plant communities.

## Introduction

The relationship between plant communities and their environment is a crucial subject in plant ecology (Soliveres et al., 2015) that changes community structure and biodiversity, resulting in characteristic vegetation (Baldeck et al., 2013; Paker et al., 2014). In this regard, the effects of invasive species invasion and environmental factors at various scales combine to produce differences in community compositions (Baldeck et al., 2013; Havel et al., 2015; Soliveres et al., 2015). The species are introduced intentionally to transport the species having high yields of crops or accidentally as a byproduct of another process (Fofonoff et al., 2003; Gaertner et al., 2016). The species invasion has rapidly increased due to rapid trade, transportation, tourism, industry growth, technological advancements, and higher urbanization rates (Ullah et al., 2022). These factors make it easier for alien species to invade different areas and endorse pressure on the natural ecosystem that provides ecosystem services to the world’s rapidly expanding human population (Pauchard et al., 2009). Therefore, invasive species have been a significant area of study for scientists worldwide (Gaertner et al., 2016; Fan et al., 2017).

An invasive species may be able to invade and spread in a new habitat despite biotic and abiotic filters (Wilson et al., 2007). Environmental parameters of any area, notably temperature, relative humidity, day length, and geographical and topographic variables, operate as an abiotic filter for newly invading invaders (Begum et al., 2021). The filter’s efficiency is determined by how closely environmental conditions correspond to the species’ native and invaded habitats (Thuiller et al., 2005). Invasive species establishment influences environmental conditions and natural plant communities directly or indirectly, including soil nutrients and physical and chemical characteristics (Weidenhamer and Callaway, 2010). Invasive plants affect evapotranspiration, causing severe threats to sustainable water supply (Levine et al., 2003) and ecosystem services such as resources and energy (Eviner et al., 2012). Invasive species may have deleterious impacts on native populations via several mechanisms, including genetic effects, pathogen introduction, and habitat degradation (Lee, 2002; Alpert, 2006).

Invasive species alter soil nutrients, particularly phosphorus (P), nitrogen (N), and potassium (K), which have a significant impact on how the soil and plants interact (Sun et al., 2019; Yinglan et al., 2019). Many studies have shown a relationship between soil N, P, and K levels and the diversity of plant species, and unimodal patterns between N, P, and K have emerged as the conventional wisdom (Pausas and Austin, 2001; John et al., 2007; Merunková and Chytrý, 2012). Species richness did not significantly alter after exogenous applications of N and P to Rengen Grassland, according to (Hejcman et al., 2010). However, it is essential to note that P and K fertilizer applications significantly changed the species composition (Hejcman et al., 2010). The total NPK contents of the soil considerably encourage the establishment of non-native shrubs and plants in the Yilong Lake Basin of the Yungui Plateau in China (Cui et al., 2009). In the hilly Ili River Valley area of China, Xu et al. (2011) revealed that the levels of TNTN, TKTK, and available phosphorus (AP) in the soil had a noticeably favorable impact on the local flora variety (Xu et al., 2011). In some instances, the soil’s pH may indicate how many nutrients are present, which can change the variety or composition of the flora (Ravolainen et al., 2013). Although there is no discernible relationship between the richness of shrubs and trees and soil pH, annual herbs, perennial herbs, and semi-shrub plants rise linearly with soil pH (Heikkinen and Neuvonen, 1997). In addition, invasive species alter the soil organic carbon (SOC), N, and pH, changing community structure and composition(Zuo et al., 2012; Cardoso et al., 2013).

However, how invasive plant communities react to floristic and vegetative characteristics and environmental factors is still being determined. Several studies (McNeish & McEwan, 2016; Subedi et al., 2019; Ullah et al., 2021; Ullah et al., 2022) strongly emphasize how invasive species traits related to environmental factors and some research focuses on how invasive species affect native plant ecosystems (Maron & Marler, 2007; Beaury et al., 2020). However, the interaction of non-native communities with one another has received very little attention in the study area, where mainly the research focuses on the invasion of single species (Khan et al., 2020; Ullah et al., 2021; Khan et al., 2022; Ullah et al., 2022). Therefore, the present study focuses on the interactions between four non-native species communities with the hypothesis that hypothesized that the local environmental conditions associated with each community would provide a unique filter evident in the community floristic structure and composition of the resident species along with the disturbance and increases or decreases of diversity. In addition, the variation in communities across the spatial and soil variables was also questioned. The four non-native communities, i.e., *Datura innoxia* (Mill.), *Xanthium strumarium* L., *Parthenium hysterophorus* L., and *Silybum marianum* L. were focused in the study because these plant species rapidly spread in the region severely affecting the native communities’ diversity. The study’s aims were 1) to assess four non-native species communities and their characteristic floristic variations, 2) to assess the environmental variables affecting these communities and evaluate diversity and similarity indices, and 3) to evaluate essential factors in maintaining four non-native species communities.

## Materials and methods

### Study area

**Figure 1:**
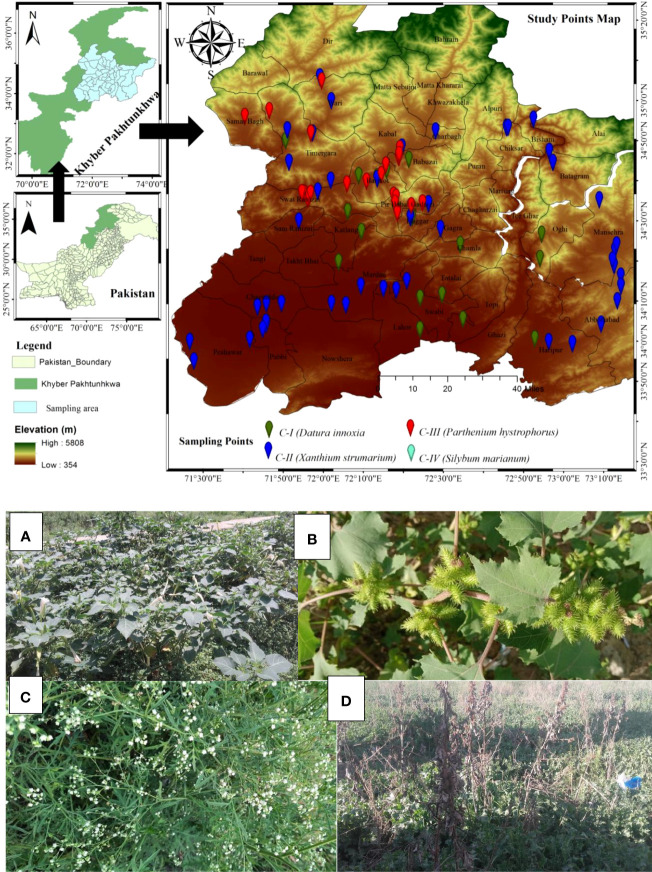
Study area map of the sampling sites representing sampling points by color Billards. Plate 1 represents four invaded communities of non-native species. **(A)** (*Datura innoxia* community); **(B)** (*Xanthium strumarium* community); **(C)** (*Parthenium hysterophorus* community); **(D)** (*Silybum marianum* community).

The study area was Pakistan’s north-west province, Khyber Pakhtunkhwa (KP), as depicted in **Figure 1**. The province boundaries are bordered by the Himalayan, Hindukush, and Karakorum mountain ranges, having a lowlands district (Peshawar) at 327 meters above sea level (asl) and uphills at an elevation of 7708 m asl (Tirch Mir Mountain peak) (Dawood, 2017). The province’s plains and mountains generate a climatic gradient varying from south to north and north to west, such as lowlands, warm climates, and cold in the northern highlands (Ali et al., 2018). Based on temperatures, the warmest month is June, having temperatures ranging from 35.01 ± 0.96°C to 18.20 ± 0.58°C, while January is the coldest month, having temperatures ranging from 14.45 ± 2.10°C to 0.83 ± 0.82°C (Shah et al., 2017), having harsher winters with freezing-point temperatures. Moreover, the relative humidity varied from 55.32 to 78.41 percent, while the yearly precipitation ranged from 379 to 743 mm (Ali et al., 2018). The area climate is vital in determining vegetation structure (Deo and Şahin, 2015). The sampled locations were between 34.59 and 34.85 N° latitudes and 360-1200 meters above sea level (**Figure 1**). The vegetation structure and communities are complicated and much dissected due to the essentially rough and changeable terrain (Ahmad et al., 2020; Ullah et al., 2021). Hence, the region sustained varied biodiverse flora and fauna (Xu et al., 2019).

### Studied species

The study considers four different non-native species communities, which are naturalized, invasive, or exotic (Plat 1). These communities were considered invaded because the non-native species were dominant in the population, i.e., having an importance value index (IVI) of 50% or > 50% for non-native species; however, in exotic species communities, the IVI may exceed more than 70% for a single species, e.g., *Silybum marianum* communities, while in some cases entire homogenized population was reported.

### Datura innoxia (Mill.)

Angel’s trumpet (*D. innoxia*) is an herbaceous perennial plant of the family Solanaceae that prefers warmer climates across the globe (tropical and sub-tropical regions). The plant grows up to 1.5 m in height and has spreading branches. The leaves are simple, glabrous, roughly oval, dark green, and shallowly lobed. *Datura* has trumpet-shaped flowers with a pleasant scent and a range of hues from white to yellow and light to dark purple; large, solitary, hermaphrodite; insect-pollinated flowers (Srivastava and Shukla, 2016). The specie is native to South and Central America (Waoo et al., 2014)and is recently naturalized in the region, spreading along with roadside areas, walls, and arable fields, disrupting the native plant ecosystems (Abeed et al., 2022).

### *Xanthium strumarium* L.

Rough cocklebur (*X. strumarium*), an annual herb of the Asteraceae family, which grows to a height of approximately 1.5 m, has a tap root and is typically reproduced by seeds (Chikuruwo et al., 2017). The visible morphological feature is oval, triangular alternating leaves, a hairy stem, and a racemose inflorescence with pistillate heads below the staminate inflorescence (Löve and Dansereau, 1959). It is a physiological short-day plant that needs 10 hours of photoperiods in the south and 7.5 hours in the north to blossom (Abdulrahman and Winstead, 1977). The specie originated from Central or South America, progressively spreading toward North America, occupying shorelines and railway tracks and dispersing widely via water and animals (Ullah et al., 2022). In KPKP, Pakistan, the specie invaded via Afghan refugee moment and pronounced impacted maize, soybean, ground nut, and cotton crops (Hashim et al., 2013). Growing as a weed, especially in soybeans and cotton, the fruits (spiny bur) are up to 2 cm long with two stouts at the end and two achenes (Hicks, 1971). As a result, the output of both soybeans and cotton is drastically reduced (up to 70%) (McWhorter and Anderson, 1976).

### *Parthenium hysterophorus* L.

Bitterweed (*P. hysterophorus*), also known as white top and bitter weed, is regarded as noxious due to its large number of seeds per plant (roughly 25,000), ability to spread quickly (Haseler, 1976), fierce competition with crops (Khosla and Sobti, 1981; McFadyen, 1992; Asif et al., 2017), and potential health risks for both humans and animals (Chippendale and Panetta, 1994). The species is considered native to Central South America or the Gulf of Mexico (Jabeen et al., 2015), is considered a noxious weed of national importance, and has rapidly spread in the arid and semi-arid environment of KPKP, Pakistan (Ali et al., 2018). The primary cause of the widespread and gregarious expansion of invasive exotic *P. hysterophorus* is human habitat disruption, which creates empty spaces that can be quickly occupied. *P. hysterophorus* has been identified as a possible significant weed of Pakistan’s natural and agricultural ecosystems because of its ability to thrive in semi-arid climates (Adkins and Navie, 2006).

### *Silybum marianum* L.

Milk thistle (*S. marianum*), of the Asteraceae family, is an annual or biennial plant widely introduced outside its native range (southeast coast of England), becoming an invasive weed in North America, Iran, Australia, and New Zealand(Abenavoli et al., 2018). It is gorgeous in flower and vegetable seed packs (Mishra et al., 2013). Once established, the milk thistle turns into a competitor for resources and water, growing in large, thick patches that shade out other plants (Berner et al., 2002). The species is considered ruderal or weedy due to its tendency to spread in patches along roads and waste areas (Gabay et al., 1994). Milk thistle became a severe weed for winter crops, including Berseem clover (*Trifolium alexandrinum* L.), Wheat (*Triticum aestivum* L.), Sugar cane (*Saccharum officinarum* L.), Oat (*Avena sativa* L.), and Barley (*Hordeum vulgare* L.) in KPKP, Pakistan (Marwat et al., 2006).

### Phytosociological data assessment

The phytosociological assessment (Communities floristics composition and classification) was entailed in 15 districts (**Figure 1**), where 21 sites of *D. innoxia*, 45 of *X. strumarium*, 24 of *P. hysterophorus*, and 75 of *S. marianum* were randomly sampled to gather vegetation data. The data set consists of 21 stands and 210 plots (21 × 10 = 210 plots), 45 stands and 450 plots (45 × 10 = 450 plots), 24 stands and 240 plots (24 × 10 = 240 plots), and 75 stands and 750 plots (75 × 10 = 750 plots) phytosociological stands and plots for *D. innoxia*, *X. strumarium*, *P. hysterophorus*, and *S. marianum*, respectively that were taken for a reasonable survey of the whole research region. We designated the *D. innoxia* community as a community I (C-I), the *X. strumarium* community as community II (C-II), the *P. hysterophorus* community as community III (C-III), and the *S. marianum* community as community IV (C-IV) for simplicity and to prevent repeating the binomial. Plot sizes were kept between 3-5 m^2^, and changes up to a factor of five were considered acceptable (Morsdorf et al., 2010). Following (Curtis and Mcintosh, 1950), the Importance value index (IVI) was calculated using relative frequency, density, and cover, in each stand. A 10-m border area from the stand’s edges was eliminated to minimize edge effects (Martínez-Falcón et al., 2018). The plant species were identified following Flora of Pakistan (Ali and Qaiser, 1986) and the naming of plant taxa according to Aeschimann et al. (2004). The number of species, cover, and density were assessed to evaluate species diversity and invasion impacts. Based on species density, the following three crucial ecological diversity indices were calculated, i.e., species richness (S), Shannon-Wiener diversity index (H′), and evenness index (E). The numeral of species in the stand was calculated to define the species richness. The following equations were used to determine the H′ and E (Equations I and II) as described by Strong (2016):


(Equation I) $$H^{\prime }=-\sum_{i=1}^{s}pi\text{ I}npi$$

(Equation II) $$E=\frac{H^{\prime }}{\text{In}S}$$

Where *pi* (species proportion), *i* (total species), In (*pi*) (natural logarithm of species proportion), and In(S) (species richness natural logarithm).

Moreover, community dissimilarities and similarities indices were calculated to evaluate the floristic and vegetation differences better. The Sørensen similarity coefficient and dissimilarity coefficient were calculated using equations III and IV following Erenso et al. (2014)


(Equation III) $${\text{S}}_{\text{S}}=2\text{a}/(2\text{a}+\text{b}+\text{c})$$

Where S_S_ is the Sørensen similarity coefficient,

a = common species in both stands,

b = unique/uncommon species in the first stand, and

c = unique/uncommon species to the second strand

The dissimilarity index can be calculated following Erenso et al. (2014)


(Equation IV) $${\text{D}}_{\text{S}}=1.0-{\text{S}}_{\text{S}}$$

### Soil analysis

The soil characteristics of each invaded stand were assessed by auger-bored 3 kg sample from the center and two opposing corners of each stand. The topsoil layer often contains a high concentration of nutrients. Therefore, samples were collected between 0 and 30 cm deep (Castillón et al., 2015), bulked, and mixed well to obtain a homogenous soil mixture (Kamrani et al., 2011; Martínez-Falcón et al., 2018). In the field, the pH was measured using a digital pH meter using a soil-water solution (1:5), and electrical conductivity (ECEC) was assessed using a conductivity meter. The soil’s physiochemical properties and textural characteristics (Sand, silt, and clay percentage) were determined using air-dried samples by running them through a 2-mm filter (Reis et al., 2015). The Walkley-Black method was used to determine the organic matter (Nelson and Sommers, 1996), while the micro-Kjeldahl method was used to assess nitrogen (%). Then, exchangeable potassium (K^+^) and phosphorus (P^2+^) in mg/Kg were calculated (Yadav et al., 2002). Geometric measurements of CO_2_ evolution and calcium carbonate (%) were measured following (Reis et al., 2015). The online calculator (https://www.nrcs.usda.gov) was used to evaluate soil hydraulic characteristics, including field capacity (FCFC), available water (AWAW), bulk density (BD), and wilting and saturation point (WSP) by following (Yadav et al., 2002). The hydrometer technique at Agriculture Research Institute (ARI) Swat assessed textural characteristics, i.e., sand, silt, and clay percentage (Gangwar and Baskar, 2019).

### Data analysis

The relative phytosociological attributes were converted into an importance value index (IVI) (Hailu, 2017) to assign each species a specific position in the four non-native communities. In addition, PC-ORD ver. 6.0, were used for further categorization of vegetation communities and was assessed using Ward’s agglomerative approach by selecting Euclidean distance (McCune, 1997). After the numerical categorization based on IVI, each species in each community was given a phytosociological category if a particular species might be regarded as dominant in a community and of a group in each community (Curtis and Mcintosh, 1950; Nabi et al., 2012; Khan et al., 2013; Biondi et al., 2015; Nasrullah et al., 2015). Each community’s environmental and soil variables and diversity indices (**Table 1**) were subjected to descriptive statistics and analysis of variance (ANOVA) to assess the variation among the non-native invaded communities. The ANOVA results were further tested by *Post hoc* (Tukey Honestly significant difference) test to evaluate the inter-communities variations. Canonical correspondence analysis (CCA) assessed the relationship between floristic variation within invaded species-dominated vegetation and environmental factors following Ali et al. (2022). Fifteen soil parameters, four environmental variables, and three types of diversity indices were utilized in the CCA, and a Monte Carlo permutation test was utilized to examine the *post hoc* interpretation of the ordination axes. The ordination bi-plots showed vector representations of the essential variables (Legendre & Gallagher, 2001). The figure was drawn using Graph Pad Ver. 7.0 (Barua et al., 2020).

**Table 1:** Environmental and soil variables and diversity indices during the analysis of communities.

Variable name	Code/Unit	Variable name	Code/Unit	Variable name	Code/Unit
Elevation	Elev./(m)	Organic matter	OM/(%)	Bulk density	BD/(%)
Latitude	Lat./(°C)	Calcium carbonate	CaCO3/(%)	Saturation point	SP/(%)
Longitude	Long./(°C)	Nitrogen	N (%)	Available water	AW/(%)
Aspect angle	AA/(°C)	Phosphorus	P/(mg/kg)	Species richness	S
Clay	CL/(%)	Potassium	K/(mg/kg)	Shannon-Wiener index	H’
Silt	SL/(%)	Electrical Conductivity	EC/(µS/cm)	Evenness index	E
Sand	SN/(%)	Wilting Point	WP/(%)		
pH	pH/(1:5)	Field Capacity	FC/(%)		

## Results

**Figure 2:**
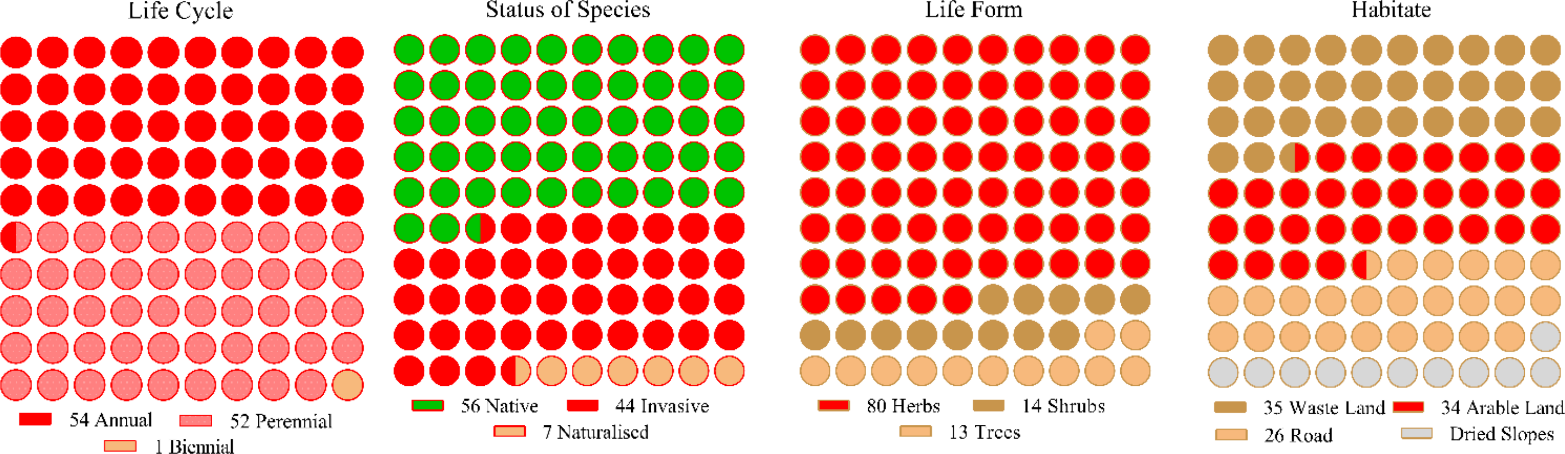
Floristic characteristics of the communities (C-I to C-IV).

In 165 sample sites, 107 species were identified, each with unique floristic features (**Figure 2**). Annual and biannual species dominated communities, each contributing 50% (54) and 49% (52) based on life cycle distribution. Similarly, native and invasive species were extensively dispersed in the communities, i.e., native (52%) and invasive (41%). In contrast, herbaceous life forms comprised 75% of the species in the communities, while the rest were shrubs and trees. Among the four communities, the species’ habitat-wise distributions included wasteland (33%), arable land (32%), roadside (23%), and dry slop (11%). Most of the plant species found in the four communities belonged to Asteraceae, Fabaceae, Lamiaceae, Solanaceae, and Amaranthaceae (**S Table 1**). The floristic comparisons of the four non-native communities are presented in **Table 2**. The highest variations in communities’ comparison were presented by the status of the communities having P< 0.001 for χ^2^ statistics. The associated plant invasive species were dominated in C-IV, i.e., 59% (13 species), followed by C-II, having 44% weightage. In contrast, the C-I and C-III were dominated by native species having 59% and 60% weightage of the community species. In life forms, the four non-native communities were dominated by herbaceous species having significant variation with χ^2^ value of 24.49 and P< 0.05. Similarly, the communities show significant differences based on the life cycle duration, having a χ^2^ value of 7.02 and P< 0.05. The annual species dominated C-I, II, and IV, while perennial species dominated C-III. Based on the C-I, II, and III habitats, the species was primarily found in roadside areas, followed by wasteland and arable land. However, in C-III, most species were inhabited by arable land and wasteland.

**Table 2:** Comparison of floristic characteristics (Status, life forms, Life cycle, and habitat) of the four non-native communities.

Parameter	Community	C-I	C-II	C- III	C-IV	CV %
Status	Native	22	17	37	4	68.19
	Naturalized	7	11	1	5	69.39
	Invasive	9	22	24	13	42.15
χ2 = 24.49, P= 0.0004		64.30	33.05	88.21	67.27	CV%
Life form	Herbs	30	44	46	14	44.24
	Shrubs	6	3	7	8	36.00
	Trees	2	3	9	0	110.66
χ2 = 24.49, P = 0.017		119.56	142.03	106.27	95.78	CV%
Life Cycle	Annual	23	31	28	16	26.77
	Biennial	1	1	1	0	66.67
	Perennial	14	18	33	6	63.80
χ2 = 7.02, NS		87.32	90.27	83.30	110.22	CV%
Habitat	Roads	16	17	10	9	31.40
	Wastelands	13	16	19	8	33.50
	Dry slop	1	6	8	5	58.88
	Arable land	8	11	25	0	94.77
χ2 = 21.35, P = 0.011		6903	40.53	51.21	73.48	CV%

The non-native species communities were categorized into four ecologically valuable groupings (C-I, C-II, C-III, and C-IV) based on dominant species in terms of IVI (**Table 3**). Notably, the second-most dominant species in each of the four communities differ uniquely, except for C-I and II, which are comparable but somewhat different in IVI, i.e., 5.72 ± 0.34 and 7.91 ± 0.88, respectively, for *P. hysterophorus*. Moreover, *D. innoxia*, with a codominance of *C. sativa* (17.28%) and *X. strumarium* (5.47%), characterizes the floristic assemblage in C-I. However, invasive *P. hysterophorus* (5.27%) and *Chenopodium album* (3.45%) also contributed to this community. The C-II was dominated by invasive *X. strumarium* (45.33%), having co-dominant species, i.e., *D. innoxia* (5.14%) and *P. hysterophorus* (7.91%), as well as numerous additional new species from their previous and following communities (C-I, III, and IV). *P. hysterophorus* (46.77%) dominates the C-III based on IVI dominance (**Table 3**). Apart from *C. dactylon* (16.72%), none of the species had IVI higher than 5% in this community. Interestingly, the group IV vegetation consisted of *S. marianum* (76.30%) with *S. nigrum* (1.75%) and *A. sativa* (1.81%).

**Table 3:** Importance Values (Mean ± SE) of individual species in a community type were computed in the analysis.

Species name	C-I	C-II	C-III	C-IV
*Datura innoxia* (Mill.)	39.04 ± 0.40	5.14 ± 0.90	0.06 ± 0.01	***
*Xanthium strumarium* L.	5.47 ± 0.39	45.33 ± 0.98	2.50 ± 0.11	***
*Parthenium hysterophorus* L.	5.27 ± 0.34	7.91 ± 0.88	46.77 ± 0.71	0.61 ± 0.03
*Silybum marianum* (L.) Geartn	0.68 ± 0.11	2.073 ± 0.61	***	76.30 ± 0.40
*Acacia nilotica* (L.) Willd. ex Delile	***	0.22 ± 0.15	0.35 ± 0.07	***
*Achyranthes bidentata* Blume.	***	***	0.06 ± 0.01	***
*Achyranthus aspera* L.	***	0.12 ± 0.12	***	***
*Ailanthus altissimus* (Mill.) Swingle	***	***	0.06 ± 0.01	***
*Ajuga bracteosa* Wall. ex Benth.	0.26 ± 0.06	***	***	***
*Alternanthera pungens* Kunth	0.28 ± 0.06	0.68 ± 0.26	***	***
*Amaranthus caudatus* L.	***	***	2.21 ± 0.17	***
*Amaranthus spinosus* L.	***	0.23 ± 0.16	***	***
*Amaranthus viridis* L.	2.32 ± 0.17	3.18 ± 0.58	***	1.05 ± 0.04
*Artemisia scoparia* Waldst. & Kit.	***	***	***	0.38 ± 0.02
*Asphodelus tenuifolius* Cav.	***	***	***	0.95 ± 0.03
*Avena fatua* L.	***	***	***	0.98 ± 0.04
*Avena sativa* L.	***	***	***	1.81 ± 0.05
*Brassica campestris* L.	***	0.43 ± 0.23	0.51 ± 0.08	***
*Brossentia papyrifera* (L.) L’Herit. ex Vent	***	0.30 ± 0.18	***	***
*Calendula arvensis* L.	3.42 ± 0.36	***	0.39 ± 0.08	***
*Calotropis procera* (Aiton) R. Br.	1.33 ± 0.16	0.52 ± 0.27	0.13 ± 0.03	***
*Cannabis sativa* L.	17.28 ± 0.52	11.89 ± 0.96	5.99 ± 0.25	0.84 ± 0.03
*Capsella bursa-pastoris* Moench	***	0.62 ± 0.31	0.17 ± 0.03	***
*Carthamus oxycantha* Bieb	1.62 ± 0.21	1.62 ± 0.53	1.07 ± 0.17	0.81 ± 0.03
*Cassia occidentalis* L.	***	0.18 ± 0.12	***	***
*Cenchrus ciliaris* L.	***	***	1.54 ± 0.21	***
*Centaurea cyanus* L.	0.32 ± 0.07	0.17 ± 0.17	***	***
*Chenopodium album* L	3.45 ± 0.28	4.11 ± 0.65	2.10 ± 0.15	1.54 ± 0.04
*Chrozophora tinctoria* (L.) Raf.	0.99 ± 0.13	0.19 ± 0.13	***	***
*Cirsium arvense* (L.) Scop.	0.34 ± 0.07	0.24 ± 0.17	***	***
*Convolvulus arvensis* L.	***	***	0.77 ± 0.15	***
*Cortaderia selloana* (Schult. & Schult.f.) Asch. & Graebn.	***	***	***	0.99 ± 0.04
*Cucurbita pepo* L.	***	0.10 ± 0.10	***	***
*Cynodon dactylon* (L.) Pers.	0.65 ± 0.09	3.74 ± 0.70	16.72 ± 0.45	***
*Cyperus rotundus* L.	***	***	0.20 ± 0.04	***
*Cyprus rotundus* L.	***	0.39 ± 0.18	***	***
*Desmostachya bipinnata* (L.) Stapf	***	***	0.32 ± 0.06	***
*Dichanthium annulatum* (Forssk.) Stapf	***	***	0.57 ± 0.07	***
*Dodonaea viscosa* (L.) Jacq.	0.25 ± 0.06	***	0.31 ± 0.06	0.88 ± 0.04
*Dysphania ambrosioides* L.	***	0.64 ± 0.31	***	***
*Echornia cresipes* (Mart.) Solma in DC	***	0.20 ± 0.20	***	***
*Eclipta alba* L.	***	0.30 ± 0.21	***	***
*Erigeron canadensis* (L.) Cronq.	***	1.30 ± 0.45	3.81 ± 0.35	***
*Eryngium caeruleum* Gilib.	1.27 ± 0.15	0.24 ± 0.18	0.38 ± 0.08	***
*Eucalyptus camaldulensis* Dehnh.	***	***	0.87 ± 0.10	***
*Euphorbia helioscopia* Dumort.	***	***	0.09 ± 0.02	1.23 ± 0.04
*Euphorbia hirta* L.	***	***	0.07 ± 0.01	1.23 ± 0.04
*Euphoria prostrate* Aiton	***	***	0.99 ± 0.14	***
*Ficus carica* L.	***	***	0.17 ± 0.02	***
*Fragaria indica* Andrews	***	***	0.04 ± 0.01	***
*Fumaria indica* Hausskn.	***	***	***	1.53 ± 0.04
*Gymnosporia royleana* Wall. ex M.A. Laws	0.34 ± 0.08	***	***	***
*Hackelia virginiana* L.	***	***	0.66 ± 0.14	***
*Helianthus annus* L.	0.29 ± 0.04	0.064 ± 0.064	***	***
*Heliotropium curassavicum* L.	0.20 ± 0.04	0.30 ± 0.25	***	***
*Hibiscus mutabilis* L.	***	***	0.09 ± 0.02	***
*Hypochaeris radicata* L.	***	***	***	1.01 ± 0.04
*Indigofera gerardiana* Graham	***	***	0.08 ± 0.02	***
*Justicia adhatoda* L.	***	0.47 ± 0.33	***	***
*Lathyrus sativus* L.	***	***	***	0.97 ± 0.04
*Lipidium sativa* L.	***	0.72 ± 0.31	***	***
*Mangifera indica* L.	***	***	0.06 ± 0.01	***
*Medicago sativa* L.	2.58 ± 0.30	***	0.20 ± 0.03	***
*Melia azedarach* L.	0.67 ± 0.10	***	0.14 ± 0.03	***
*Melilotus indicus* (L.) All.	***	***	***	1.73 ± 0.05
*Mentha longifolia* (L.) Huds.	***	0.13 ± 0.13	1.15 ± 0.15	***
*Mentha spicata* L.	***	***	0.14 ± 0.02	***
*Mirabilis jalapa* L.	1.23 ± 0.16	0.39 ± 0.29	0.23 ± 0.03	***
*Morus alba* L.	0.71 ± 0.12	***	0.43 ± 0.04	***
*Narcissus tazetta* DC.	***	***	0.08 ± 0.02	***
*Nasturtium officinale *W.T. Aiton	***	***	0.15 ± 0.02	***
*Origanum vulgare* (Linn)	0.55 ± 0.08	***	***	***
*Otostegia lambata* Benth.	0.66 ± 0.14	***	***	***
*Oxalis carniculata* L.	1.72 ± 0.24	0.073 ± 0.07	2.23 ± 0.15	***
*Persicaria maculosa* S.F.Gray	0.32 ± 0.07	2.07 ± 0.58	0.11 ± 0.02	***
*Phalaris caroliniana* Walter	***	***	***	0.66 ± 0.03
*Phalaris minor* Retz.	***	***	***	1.65 ± 0.05
*Phragmites karka* (Retz.) Trin. ex Steud.	***	***	0.13 ± 0.02	***
*Physalis minima* L.	***	0.18 ± 0.12	***	***
*Pinus roxburghii* Sarg.	***	***	0.19 ± 0.02	***
*Poa annua* Fr. ex Andersson	***	***	0.20 ± 0.04	***
*Populus nigra* L.	***	***	0.23 ± 0.05	***
*Prosopis julifolia* (Sw.) DCDC.	***	0.12 ± 0.12	***	***
*Ricinus communis* L.	2.32 ± 0.19	***	***	***
*Robinia pseudoacacia* L.	***	***	0.73 ± 0.13	***
*Rubus fruticosus* L.	***	***	0.11 ± 0.02	***
*Rumex dentatus* L.	***	***	1.34 ± 0.10	***
*Rumex hastatus* D.Don	0.49 ± 0.07	***	0.15 ± 0.03	***
*Salvia moorcroftiana* Wall. ex Benth.	0.43 ± 0.06	***	0.14 ± 0.02	***
*Solanum melongena* (Mill.) Dunal	***	***	0.04 ± 0.01	***
*Solanum nigrum* L.	0.70 ± 0.09	0.31 ± 0.15	0.57 ± 0.06	1.75 ± 0.05
*Solanum xanthocarpum* Schrad. & J.C. Wendl.	***	***	0.06 ± 0.01	***
*Sonchus asper* L.	***	0.60 ± 0.24	0.06 ± 0.01	***
*Spergula arvensis* L.	***	***	***	1.22 ± 0.04
*Stylosanthes humilis* Kunth	***	***	***	1.10 ± 0.04
*Tagetes erectus* L	***	0.72 ± 0.56	***	***
*Tagetes minuta* L.	***	0.53 ± 0.53	0.54 ± 0.07	***
*Taraxicum officinale* Weber	***	0.58 ± 0.28	***	***
*Trianthema portulacastrum* L.	0.36 ± 0.08	0.11 ± 0.11	***	***
*Tribulus terrestris* L.	0.58 ± 0.13	0.58 ± 0.43	0.15 ± 0.03	***
*Trifolium repens* L.	***	1.05 ± 0.47	***	***
*Utrica dioica* L.	***	0.18 ± 0.13	***	***
*Verbascum Thapsus* L.	0.18 ± 0.04	0.94 ± 0.44	0.19 ± 0.02	***
*Zanthoxylum armatum* DCDC.	0.78 ± 0.17	***	***	***
*Zea mays* L.	***	0.087 ± 0.087	0.04 ± 0.01	***
*Ziziphus nummularia* Aubrév.	***	***	0.05 ± 0.01	***
*Ziziphus oxyphylla* Edgew.	***	***	0.05 ± 0.01	***

*** represents absence of a species in particular community.

**Figure 3:**
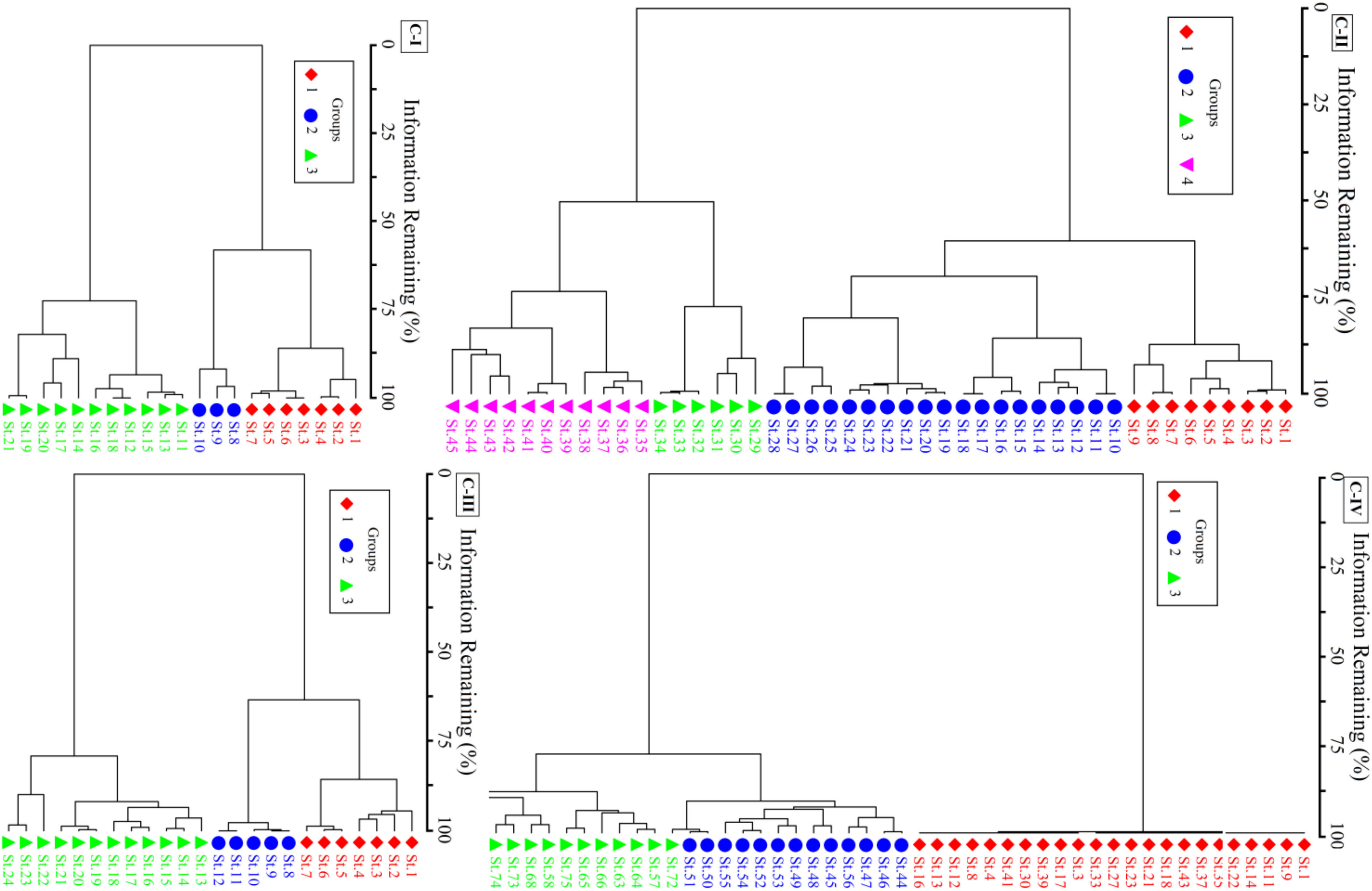
Ward’s agglomerative cluster classifying the communities C-I to C-IV.

The four non-native communities were further categorized using Ward’s agglomerative cluster into groups (**Figure 3**). C-I consists of three groups having *P. hysterophorus* co-dominant species of group I. In contrast, invasive species might be categorized as part of group II, i.e., the *Datura-Parthenium-Cannabis* community. The co-dominant species of group III was *Medicago denticulate*, also known as the *Datura-Medicago* group. This native species shows that group III is more stable than groups I and II. The C-II, *X. strumarium* invaded regions were divided into four ecologically significant groups based on different ecosystems and species composition. I was distinguished by the presence of *Xanthium-Cannabis*, with *C. album* and *C. dactylon* predominating. The overwhelming prevalence of invasive *P. hysterophorus* and *C. album* in group II set it apart. In contrast to group IV, this includes *X. strumarium*-*C*. *sativa*, group III has *X. strumarium-D. innoxia*. The co-dominant specie between groups I and IV was similar, but since additional distinctive species are present, they are considered separate groups. Three separate groups comprise the C-III of *P. hysterophorus*, with group-I being dominated by *P. hysterophorus* and two significant related species, namely *C. sativa* and *C. dactylon*. Similar to group I, groups II and III likewise have *C. dactylon* as the co-dominant species. In C-IV, most stands had a pure population of *S. marianum*, which was grouped as group-I in the cluster dendrogram. Nevertheless, three co-dominant species, namely *Phalaris minor*, *Stylosanthes humilis*, and *Fumaria indica*, were in the heavily invaded group (group II). *Melilotus indicus*, *Solanum nigrum*, and *Euphorbia helioscopia* were the three co-dominant species in partially invaded communities, i.e., group III.

### Communities’ diversity and environmental variables

**Figure 4:**
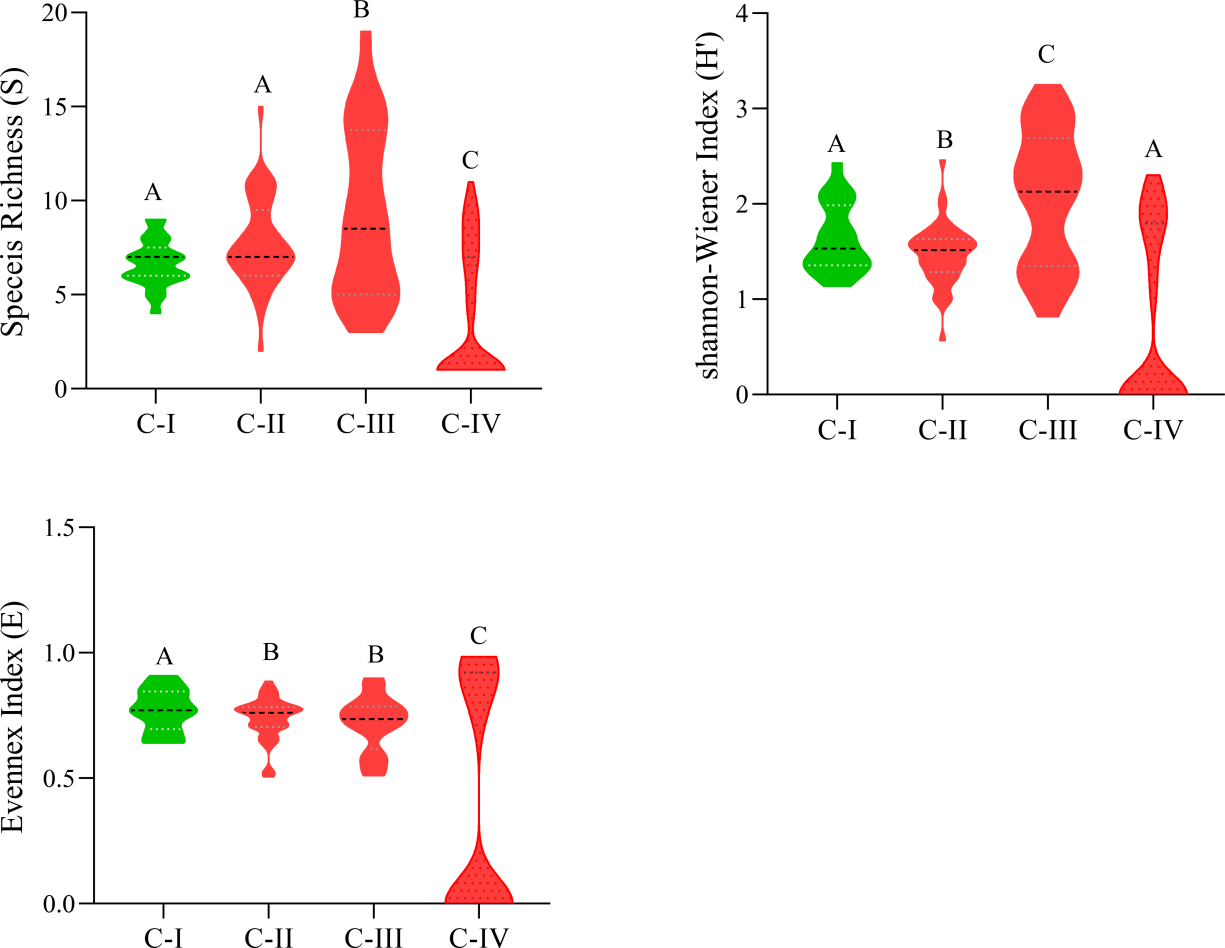
Species diversity indexes of C-I to C-IV communities. Different letters in the columns (A and B) depict significant differences (P< 0.05).

C-I has the lowest species richness, i.e., nine species/stand, while C-III has diverse stands having higher species richness, i.e., nineteen species/stand. The Shannon-Wiener diversity index changes considerably (p< 0.05), from 2.06 ± 0.71 (C-III) to 0.74 ± 0.91 (C-IV). The species evenness index, in contrast, varies in marginal ranges (0.77 ± 0.08 (C-I)-0.37 ± 0.45 (C-IV), showing considerable variance (P< 0.05) as depicted in **Figure 4**. Similarly, Sorenson similarity and dissimilarity indices were calculated as presented in **Table 4**. The highest similarity was shown between C-I and C-II with a Sorenson similarity index of 0.55, while the lowest was between C-I and C-IV (0.13).

**Table 4:** Similarity and dissimilarity indices of the four communities.

Sorenson (Czekanowski) Similarity (Ss)					Sorenson dissimilarity index			
Community	C-I	C-II	C-III	C-IV	C-I	C-II	C-III	C-IV
C-I	1				0			
C-II	0.55	1			0.45	0		
C-III	0.45	0.45	1		0.55	0.55	0	
C-IV	0.13	0.15	0.19	1	0.87	0.85	0.81	0

In environmental factors, latitude, longitude, and aspect angle were the most prominent factor that differed significantly across communities (P< 0.001). The C-III lies at a higher elevation of 803.42 ± 7.05 m, whereas C-IV stands at 675.32 ± 7.12 m, showing non-significant variation. Similarly, in soil texture, C-IV had a higher percentage of silt (32.43 ± 0.11) than C-I, II, and IV, with a significant difference indicated by ANOVA (F-value 6.24 and P-value 0.0005). The organic matter in C-I was lower (1.29 ± 0.04%), whereas higher in C-III (2.22 ± 0.04%) than in II and IV; however, it differed non-significantly (F-value 0.24 and P-value 0.86). Likewise, C-IV had the highest CaCO_3_ percentage mean value (11.51 ± 0.05) compared to the other communities. In contrast, the C-III had the highest CaCO_3_ percentage (0.56 ± 0.01), indicating a significant difference demonstrated by ANOVA (F-value 58.3 and P-value 1.1×10^-^). The soil of C-IV has the highest mean value of nitrogen (2.90 ± 0.13%) compared to C-I, II, and III, with a significant difference demonstrated by ANOVA (F-value 2.4 and P-value 0.05). In contrast, the soil hydraulic parameters (Wilting point, Field Capacity, Bulk density, Saturation point, and Available water) of C-I-IV vary in very narrow ranges and differ non-significantly, i.e., P > 0.05 (**Table 5**).

**Table 5:** Environmental and Soil characteristics of the C-I to C-IV communities.

Parameter	Code	C-I	C-II	C-III	C-IV	S
Elevation	Elev. (m)	1752.85 ± 7.47	716.33 ± 8.56	803.42 ± 7.05	675.32 ± 7.12	NSNS
Latitude	Lat. (°C)	134.42 ± 0.01a	34.42 ± 0.01a	34.70 ± 0.01b	34.63 ± 0.01c	***
Longitude	Long. (°C)	72.36 ± 0.01a	72.36 ± 0.01a	72.11 ± 0.01b	71.98 ± 0.003c	***
Aspect angle	AA (°C)	162.57 ± 5.02a	157.67 ± 2.28a	201.25 ± 4.77b	102.53 ± 0.59c	***
Clay	CL (%)	24.066 ± 0.46a	27.64 ± 0.24b	25.37 ± 0.46b	32.43 ± 0.11c	***
Silt	SL (%)	42.49 ± 0.92a	38.87 ± 0.36a	34.17 ± 0.43ab	35.56 ± 0.12ab	*
Sand	SN (%)	33.45 ± 0.78a	33.49 ± 0.33a	41.59 ± 0.51b	32.08 ± 0.12c	**
pH	pH (1:5)	6.73 ± 0.01a	6.81 ± 0.01a	6.90 ± 0.02b	6.08 ± 0.01c	***
Organic matter	OM%	1.29 ± 0.04	1.39 ± 0.02	2.22 ± 0.04	1.35 ± 0.09	NSNS
Calcium carbonate	CaCO_3_ (%)	8.99 ± 0.19a	8.79 ± 0.07a	0.56 ± 0.01b	11.51 ± 0.05c	***
Nitrogen	N %	0.06 ± 0.00a	0.048 ± 0.00a	0.12 ± 0.00a	2.90 ± 0.13b	*
Phosphorus	P (mg/kg)	4.91 ± 0.06a	4.82 ± 0.03a	3.41 ± 0.10b	6.63 ± 0.02c	***
Potassium	K (mg/kg)	103.14 ± 1.94a	106.33 ± 0.80a	107.17 ± 1.66b	144.07 ± 0.49c	***
Electrical Conductivity	ECEC (µS/cm)	332.76 ± 4.57a	305 ± 2.10a	757.58 ± 27.68b	433.73 ± 5.55c	***
Wilting Point	WPWP (%)	0.15 ± 0.00	0.17 ± 0.00	0.15 ± 0.00	0.15 ± 0.001	NSNS
Field Capacity	FCFC (%)	0.29 ± 0.00	0.30 ± 0.00	0.28 ± 0.00	0.29 ± 0.000	NSNS
Bulk density	BDBD (%)	1.38 ± 0.00	1.36 ± 0.00	1.39 ± 0.00	1.36 ± 0.000	NSNS
Saturation point	SPSP (%)	0.48 ± 0.00	0.49 ± 0.00	0.48 ± 0.00	0.48 ± 0.00	NSNS
Available water	AWAW (%)	0.15 ± 0.00	0.14 ± 0.00	0.13 ± 0.00	0.14 ± 0.00	NS

S (Significance level); *** (P< 0.001); ** (P< 0.01); * (P< 0.05); NS (Not Significant); Different superscripts represent statistically significant differences in ANOVA at p< 0.05.

### Communities-environment relationships

**Figure 5:**
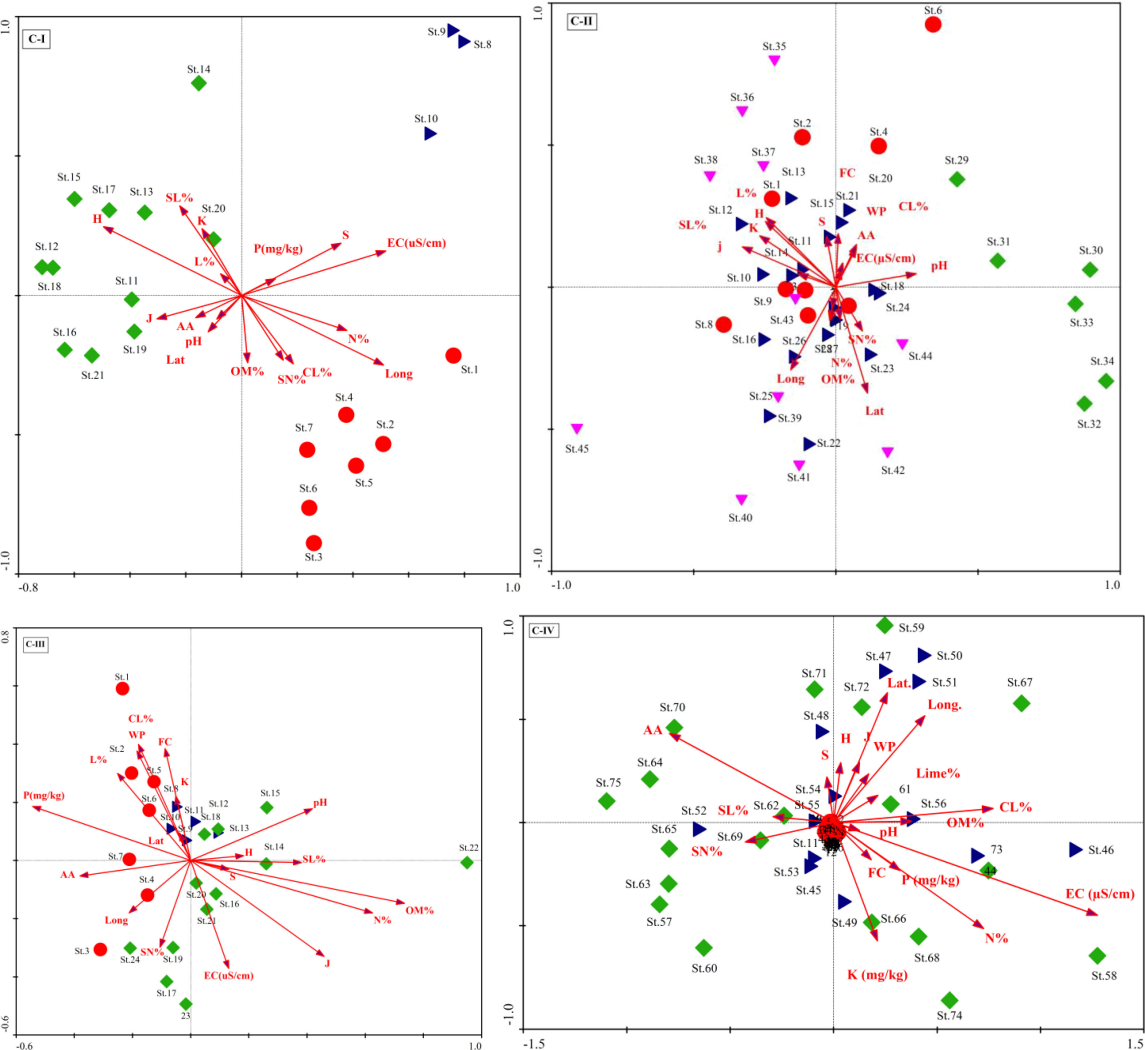
Canonical correspondence analysis (CCA) biplots for the plant communities (C-I to C-IV) and their environmental factors.

The CCA Axis 1 accounted for 16.3% of the variance in C-I, followed by axes 2 and 3 and 42.2% of the variation on the three axes (**Table A1**). The first ordination axis showed strong positive correlations with organic matter (r =0.33), the Evenness index (*r* =0.32), and the Shannon-Wiener diversity index (*r* =0.41). On the other hand, significant negative correlations exist on axis 1 for essential variables such as lime % (*r* = -0.28), silt percentage (r = -0.26), and species richness (*r* = -0.44). These factors are illustrated in the CCA biplot, represented by arrows showing their Biplot scores (**Figure 4**). The CCA analysis of C-II revealed that axis 1 has 5.9% of the total variations, followed by axes 2 and 3 with 5.8% and 5.6% variations (**Table A2**). The correlation coefficient of nutrient contents like organic matter (*r* = 0.37), and phosphorus (*r* = 0.45), were significant on axis 3. In contrast, the diversity indices, i.e., the Evenness index (*r* = 0.23) and the Shannon-Wiener diversity index (0.20), revealed significant positive relationships on axis 1. However, elevation gradient with a negative influence (*r* = -0.42) on *X. strumarium*-dominated vegetation on the first axis. In addition, clay contents showed a comparatively strong positive correlation on axes 2 and 3 (*r* = 0.41 and 0.31, respectively) and may have a substantial role in maintaining C-II (**Figure 5**).

In C-III, the CCA axes of the vegetation data explained a cumulative variance of 34.5% (13.5%, 12%, and 9%, for axes 1, 2, and 3, respectively). The first axis revealed a strong positive association (*r*=-0.41) with latitude and a negative correlation (*r* = 0.23) with longitude, while axis 2 had the strongest positive correlation (*r* = 0.41) with % clay. The findings imply that the vegetation composition is governed by the topographic gradient from lowland to highland mountains with low sand particles. In contrast, the second axis had modest connections with field capacity (r = 0.33), bulk density (*r* = 0.33), and available water (*r* = -0.32), as given in **Table A3**. The CCA in C-IV shows heavily loaded data on axis 1, with an Eigenvalue of 0.43 and a variance of 27.3, corresponding to 98% of Pearson’s correlation. The three CCA axes explained 37% variation, with axis two and axis 3 contributing 6.5 and 3.3%, respectively (**Table A4**). The ordination biplot showed a definite deflection and separation of fully invaded areas from severely and partly invaded sites. The biplot showed that environmental variables (aspect degree and elevation), nutrients (CaCO_3_%, potassium, and phosphorus), and soil texture (sand, silt, and clay) factors all had an impact on the communities (**Figure 4**).

## Discussion

In the floristics spectrum, 107 plant species were recorded in the studied region, belonging to 25 families. The Asteraceae, Amaranthaceae, Solanaceae, Brassicaceae, and Fabaceae had the most species diversity, while the other families had the least. This relationship between plant families and invading regions having low species richness has been reported globally. For instance, (Qureshi et al., 2014; Seifu et al., 2017; Iqbal et al., 2020) found that the Asteraceae, Poaceae, Amranthaceae, and Fabaceae were the dominant families of non-native species-dominated communities. Even though our data concurred with these findings, differences in taxonomic ranks at the family level may be related to geography and climate (Kosanic et al., 2018).

In the floristics composition, some of the species reported were of conservation concern, e.g., *Calotropis procera*, *Artemisia scoparia*, and *Indigofera gerardiana* were recently reported to be rare species in the region (Ilyas, 2015; Faisal and Khan, 2022). Native species become more susceptible to extinction due to non-native species disrupting the variety and composition of native plant ecosystems (Zimmermann, 1991). The ability of non-native species to alter the physical and chemical composition of local soils, in turn altering community diversity, is an essential factor that assesses its potential to become naturalized (Iqbal et al., 2021). The communities were dominated by annual and perennial plant species, which is related to the opposing theories put forth in the literature that 1) annual plants, in particular, have significant effects on the homogenization of communities (Qian and Guo, 2010) and 2) perennial plants encourage community disturbance and invasion (Milchunas et al., 1988; Tappeiner et al., 1991). However, given the propensity for bigger plants to displace smaller ones over time, little herbs may be disadvantaged (Timsina et al., 2011). In areas where herbs or shrubs predominate, invasion of certain plant species was dominant on the sides of highways and waste fields, as well as in agricultural fields. Similar predictions have been made by (Burleigh, 2005; Brisson et al., 2010; Richardson, 2011; Davies et al., 2013; Salgado et al., 2019) that agricultural fields, railroad lines, and road pavements would be the most vulnerable to plant invasion.

Low diversity indices were recorded in the study region, possibly related to the high invasion rate at the assessed sites. Several researchers, e.g., (Seifu et al., 2017; Qureshi et al., 2019; Iqbal et al., 2020; Iqbal et al., 2021), reported that invaded sites have low diversity indices. In non-native populations, Shannon diversity and IVI showed a declining tendency along invasion gradients. For instance, colonies of *S. marianum* that have been entirely or severely invaded have low diversity indices. In invaded areas, Seifu et al. (2017); Qureshi et al. (2019), and Iqbal et al. (2020) demonstrated poor diversity of vegetation in sites invaded by non-native species. However, the number of species in a community is little impacted by non-native naturalized species (Awasthi et al., 2012), such as in the communities of *D. innoxia.* Similarly, *D. innoxia* and *Cannabis sativa* are native invasive herbaceous plants with high growth rates, which may encourage a rise in native, fast-growing species due to compositional changes.

In environmental variables, elevation was a prominent factor in every community, i.e., C-I-IV, across which the vegetation characteristics vary (**S Table 2-5**). The species were most abundant at lower elevations, as high elevations have more diurnal variation in the climate (Zia et al., 2018). Along the elevation gradient in non-native communities, our results showed distinct and substantial variations in community composition across environmental variables. For instance, as the altitude rises, the percentages of OM, N, and available water decrease while the percentages of lime, silt, and lime increase. Elevation impacts soil characteristics (Blackburn et al., 2011), while (Kosanic et al., 2018) reported that a plant species’ capacity to invade the soil indirectly or directly impacts the chemical composition and availability of nutrients. According to research, invasive and naturalized species may use the nutrients in the soil to control their establishment and reproduction (Qian and Guo, 2010; Kosanic et al., 2018). The consistency of soil nutrients may also result from alien naturalized/invasive plants, which might encourage future invasions into populated regions (Qian and Guo, 2010). Carbon, nitrogen, phosphorous, and potassium increased in invaded areas (Bekele, 2000). In nutrient-rich situations, plant growth rate and biomass production increased concurrently with changes in plant biomass (Tappeiner et al., 1991), consistent with our findings. Environmental variable alterations have been shown to assist invaders like *X. strumarium* (Gallardo et al., 2017) and *P. hysterophorus* disruption (Milchunas et al., 1988), and this may also be true for *D. innoxia*. A combination of soil instability and fertilizer inputs is crucial for promoting and establishing non-native plants (Salgado et al., 2019). The relationship between plant invasion and soil nutrition is well established (Davies et al., 2013) and is shown by the increased degradation and microbiological activity in *P. hysterophorus*-infected soils (Brisson et al., 2010). *D. innoxia* has experienced the same enhancement of microbiological activities as evident from nutrient-rich zones that were primarily invaded. Based on our findings, invasion and naturalization intensities may be restricted at higher elevations, as evident from decreased non-native species IVI in higher elevation sites.

Although the precise mechanism and year of the community invasion in the area are unknown, non-native species have unintentionally been introduced into several Khyber Pakhtunkhwa regions over the past two decades during the movements of Afghan refugees, flood runoff, and tourist visits for recreational purposes (Gaertner et al., 2016; Gallardo et al., 2017). These non-native species have been widely established, naturalized, and multiplied, negatively affecting biodiversity and agriculture (Marwat et al., 2010). Depending on how well they adapt to the new environment, non-native plant species in new habitats may either grow or perish (Ullah et al., 2022). It is challenging to foresee whether these would flourish and naturalize in the new setting (Sherley, 2000; Marwat et al., 2010; Gallien and Carboni, 2017). The successful, invasive plants often spread, altering vegetation and environmental factors (Zimmermann, 1991; Le Maitre et al., 2011; Ratnayake, 2014; Ullah et al., 2022) and endangering native plants and animals (Zimmermann, 1991; Qureshi et al., 2019). Our research confirmed prior assertions that these invasive non-native plants have effectively supplanted local plant species, colonized the semi-arid parts of Khyber Pakhtunkhwa, Pakistan, and established themselves as significant invasive species there.

## Conclusions

The findings of the research suggest that the area is invaded by different types of non-native species, which may be naturalized (*D. innoxia*), invasive (*X. strumarium*), and exotic (*P. hysterophorus* and *S. marianum*). In these species, *S. marianum* form pure communities having homogenized communities. In contrast, the *P. hysterophorus* communities were diverse, consisting of various native and non-native species suggesting that invasive species can decrease or increase biodiversity. The research indicated that the communities in the area are at risk, and proper strategies are needed to control and overcome the spread of these non-native species. By conducting qualitative and quantitative monitoring using species inventories (seasonally) and phytosociological methodologies, as well as mapping using ground-based techniques (through map overlays or GPS) and remotely sensed images (aerial photos, high-resolution multispectral digital data), future invasions of such invasive species may be prevented. Researchers need to engage with taxonomists, ecologists, and land managers to establish a plant detection network in vulnerable areas to prevent such species from becoming invasive. The CCA analysis of each community revealed the importance of elevation gradient in spreading these invasive species. Therefore, the environmental gradient must be considered while devising strategies for controlling these invasive species.

Moreover, the life cycle of these species in most cases was also non-overlapped, indicating the disturbance of communities in the whole growing period. In addition, there is also a need to highlight the use of these invasive species in ecosystem services as bioresources for different purposes. The biomass of invasive species may also be utilized for medicinal and phytochemical purposes, and some of these species, such as *X. strumarium*, have recently been reported for heavy metal pollution biomonitoring and phytoremediation (Ulalh and Khan, 2022). We also advise assessing these species’ morphological, phenological, phytochemical, physiological, and reproductive biology for efficient management. In addition, as suggested by Dickie et al. (2014), we argue to assess the positive and negative traits of such species be investigated in a broad, systematic framework to prevent any inconsistencies between their risks and benefits.

## Data availability statement

The original contributions presented in the study are included in the article/**Supplementary Material**. Further inquiries can be directed to the corresponding author.

## Author contributions

NK, and RU conceptualization this research, conducted the field and laboratory experiments, and compiled and analyzed the data; NK and RU wrote the original draft of the manuscript; MO, and MA-M supervised the experiments; MO, and MA-M, contributed to data analysis, helped in review and edited the initial draft of the manuscript; all authors read and approved the manuscript.
