# Supplementary material for: Spatial distribution of the four invasive plants and their impact on natural communities’ dynamics across the arid and semi-arid environments in northwest Pakistan
Source: Front Plant Sci. 2023 Aug 25;14:1207222. doi: 10.3389/fpls.2023.1207222 (PMC10485558; doi:10.3389/fpls.2023.1207222)
Supplement: Supplementary file 1 [file DataSheet_1.docx]

A Table 1: Axes summary, correlation and biplot scores of the of the environmental and climatic variables affecting the C-I groups

| Axis | | | | Axis 1 | Axis 2 | Axis 3 |
| --- | --- | --- | --- | --- | --- | --- |
| Eigenvalue | | | | 0.474 | 0.402 | 0.354 |
| Data Variance | | | | | | |
| Percentage variance | | | | 16.3 | 13.8 | 12.2 |
| Cumulative variance | | | | 16.3 | 30.1 | 42.2 |
| Spp-Env Pearson Correlation | | | | 0.984 | 0.993 | 0.976 |
| Spp-Eniv (Rank) Corr. | | | | 0.895 | 0.781 | 0.829 |
| Correlation | | | | Biplot Scores | | |
| Code | Axis 1 | Axis 2 | Axis 3 | Axis 1 | Axis 2 | Axis 3 |
| Elev. (m) | -0.09 | -0.66 | -0.242 | -0.062 | -0.419 | -0.144 |
| Lat. (◦) | 0.291 | 0.12 | -0.14 | 0.2 | 0.076 | -0.083 |
| Long. (◦) | -0.172 | 0.588 | -0.108 | -0.119 | 0.373 | -0.064 |
| AA (◦) | 0.107 | 0.008 | 0.092 | 0.074 | 0.005 | 0.055 |
| CL (%) | 0.129 | 0.232 | -0.028 | 0.089 | 0.147 | -0.017 |
| SL (%) | -0.265 | -0.441 | -0.045 | -0.182 | -0.28 | -0.027 |
| SN (%) | 0.236 | 0.382 | 0.07 | 0.162 | 0.243 | 0.042 |
| pH (1:5) | 0.083 | -0.137 | 0.379 | 0.057 | -0.087 | 0.225 |
| OM% | 0.337 | 0.249 | -0.258 | 0.232 | 0.158 | -0.154 |
| CaCO_3_ (%) | -0.284 | -0.126 | -0.004 | -0.196 | -0.08 | -0.002 |
| N % | -0.071 | 0.463 | -0.115 | -0.049 | 0.294 | -0.069 |
| P (mg/kg) | 0.064 | 0.176 | -0.366 | 0.044 | 0.112 | -0.218 |
| K (mg/kg) | 0.009 | -0.048 | -0.305 | 0.006 | -0.03 | -0.182 |
| EC (µS/cm) | -0.285 | 0.374 | -0.436 | -0.196 | 0.237 | -0.259 |
| BD (%) | -0.019 | 0.057 | 0.042 | -0.013 | 0.036 | 0.025 |
| SP (%) | 0.014 | -0.058 | -0.048 | 0.01 | -0.037 | -0.028 |
| AW (%) | -0.306 | -0.267 | -0.219 | -0.211 | -0.17 | -0.13 |
| S | -0.443 | 0.072 | 0.059 | -0.305 | 0.046 | 0.035 |
| H | 0.413 | -0.328 | -0.37 | 0.284 | -0.208 | -0.22 |
| J | 0.328 | -0.009 | -0.193 | 0.226 | -0.005 | -0.115 |

Note: Variables codes were the same as that of Table 4 and Figure 4

A Table 2: Summary statistics, Intra-set correlations and biplot scores for the first three axes obtained from CCA-ordination of C-II groups

| Axis | | | | Axis 1 | Axis 2 | Axis 3 |
| --- | --- | --- | --- | --- | --- | --- |
| Eigenvalue | | | | 0.197 | 0.192 | 0.186 |
| Variance in species data | | | | | | |
| Percentage variance | | | | 5.9 | 5.8 | 5.6 |
| Cumulative variance | | | | 5.9 | 11.7 | 17.3 |
| Spp-Env Pearson Correlation | | | | 0.923 | 0.92 | 0.919 |
| Spp-Eniv (Rank) Corr. | | | | 0.696 | 0.715 | 0.737 |
| Code | Correlation | | | Biplot Scores | | |
|  | Axis 1 | Axis 2 | Axis 3 | Axis 1 | Axis 2 | Axis 3 |
| Elev. (m) | -0.42 | 0 | 0.232 | -0.187 | 0 | 0.1 |
| Lat. (◦) | -0.02 | -0.49 | 0.119 | -0.007 | -0.216 | 0.051 |
| Long. (◦) | -0.49 | 0.303 | 0.462 | -0.218 | 0.133 | 0.199 |
| AA (◦) | -0.14 | -0.07 | -0.08 | -0.064 | -0.032 | -0.035 |
| CL (%) | 0.199 | 0.411 | 0.315 | 0.088 | 0.18 | 0.136 |
| SL (%) | -0.24 | 0.018 | -0.35 | -0.108 | 0.008 | -0.151 |
| SN (%) | 0.124 | -0.32 | 0.156 | 0.055 | -0.142 | 0.067 |
| pH (1:5) | 0.281 | 0.171 | 0.539 | 0.125 | 0.075 | 0.232 |
| OM% | -0.11 | 0.169 | 0.373 | -0.048 | 0.074 | 0.161 |
| CaCO_3_ (%) | 0.28 | 0.298 | -0.42 | 0.124 | 0.131 | -0.181 |
| N % | 0.051 | 0.041 | 0.044 | 0.023 | 0.018 | 0.019 |
| P (mg/kg) | 0.069 | -0.17 | 0.455 | 0.031 | -0.073 | 0.196 |
| K (mg/kg) | -0.02 | 0.537 | -0.17 | -0.007 | 0.235 | -0.071 |
| EC (µS/cm) | -0.08 | -0.13 | 0.027 | -0.037 | -0.056 | 0.012 |
| WP (%) | 0.25 | 0.408 | 0.313 | 0.111 | 0.179 | 0.135 |
| FC (%) | 0.142 | 0.48 | 0.177 | 0.063 | 0.21 | 0.076 |
| BD (%) | -0.06 | -0.43 | -0.2 | -0.025 | -0.189 | -0.084 |
| SP (%) | 0.053 | 0.43 | 0.198 | 0.024 | 0.188 | 0.085 |
| AW (%) | -0.2 | 0.168 | -0.3 | -0.088 | 0.073 | -0.128 |
| S | 0.231 | 0.025 | 0.045 | 0.102 | 0.011 | 0.019 |
| H | 0.205 | 0.14 | -0.15 | 0.091 | 0.061 | -0.064 |
| J | -0.03 | 0.154 | -0.17 | -0.015 | 0.068 | -0.074 |

Note: Variables codes were the same as that of Table 4 and Figure 4

A Table 3: Summary statistics table for the CCA ordination of C-III groups

|  | | | | Axis 1 | Axis 2 | Axis 3 |
| --- | --- | --- | --- | --- | --- | --- |
| Eigenvalue | | | | 0.406 | 0.361 | 0.272 |
| Variance in species data | | | | | | |
| Percentage variance | | | | 13.5 | 12 | 9 |
| Cumulative variance | | | | 13.5 | 25.5 | 34.5 |
| Spp-Env Pearson Correlation | | | | 0.984 | 0.998 | 0.994 |
| Spp-Eniv (Rank) Corr. | | | | 0.819 | 0.928 | 0.949 |
|  | Correlation | | | Biplot Scores | | |
| Code | Axis 1 | Axis 2 | Axis 3 | Axis 1 | Axis 2 | Axis 3 |
| Elev. (m) | 0.148 | 0.062 | 0.032 | 0.094 | 0.038 | 0.017 |
| Lat. (◦) | 0.43 | 0.125 | 0.185 | 0.274 | 0.075 | 0.096 |
| Long. (◦) | -0.233 | -0.212 | 0.233 | -0.148 | -0.127 | 0.121 |
| AA (◦) | 0.027 | 0.35 | 0.327 | 0.017 | 0.21 | 0.17 |
| CL (%) | 0 | 0.374 | 0.045 | 0 | 0.225 | 0.024 |
| SL (%) | 0.214 | -0.216 | -0.478 | 0.137 | -0.13 | -0.249 |
| SN (%) | -0.197 | -0.201 | 0.232 | -0.125 | -0.121 | 0.121 |
| pH (1:5) | 0.046 | -0.071 | -0.525 | 0.029 | -0.043 | -0.273 |
| OM% | -0.168 | -0.423 | -0.51 | -0.107 | -0.254 | -0.266 |
| CaCO_3_ (%) | 0.079 | 0.01 | 0.102 | 0.051 | 0.006 | 0.053 |
| N % | -0.04 | -0.446 | -0.415 | -0.026 | -0.268 | -0.216 |
| P (mg/kg) | 0.155 | 0.175 | 0.57 | 0.099 | 0.105 | 0.297 |
| K (mg/kg) | -0.418 | 0.327 | -0.102 | -0.267 | 0.197 | -0.053 |
| EC (µS/cm) | 0.028 | -0.303 | -0.12 | 0.018 | -0.182 | -0.063 |
| WP (%) | 0.007 | 0.356 | 0.028 | 0.005 | 0.214 | 0.014 |
| FC (%) | 0.086 | 0.335 | -0.085 | 0.055 | 0.202 | -0.044 |
| BD (%) | -0.048 | -0.355 | 0 | -0.031 | -0.213 | 0 |
| SPSP (%) | 0.049 | 0.353 | 0.003 | 0.031 | 0.212 | 0.002 |
| AW (%) | 0.253 | 0.007 | -0.324 | 0.161 | 0.004 | -0.169 |
| S | -0.18 | -0.062 | -0.126 | -0.115 | -0.037 | -0.066 |
| H | -0.216 | -0.001 | -0.157 | -0.138 | -0.001 | -0.082 |
| J | -0.126 | -0.239 | -0.433 | -0.08 | -0.144 | -0.226 |

Note: Variables codes were the same as that of Table 4 and Figure 4

A Table 4: Axis summary and commutative variance of the variables affecting C-IV groups

| Axis | | | | Axis 1 | Axis 2 | Axis 3 |
| --- | --- | --- | --- | --- | --- | --- |
| Eigenvalue | | | | 0.438 | 0.104 | 0.052 |
| Variance in species data | | | | | | |
| Percentage variance | | | | 27.3 | 6.5 | 3.3 |
| Cumulative variance | | | | 27.3 | 33.8 | 37 |
| Spp-Env Pearson Correlation | | | | 0.983 | 0.738 | 0.687 |
| Spp-Eniv (Rank) Corr. | | | | 0.665 | 0.346 | 0.44 |
|  | Correlation | | | Biplot Scores | | |
| Code | Axis 1 | Axis 2 | Axis 3 | Axis 1 | Axis 2 | Axis 3 |
| Elev. (m) | 0.851 | -0.059 | 0.115 | 0.563 | -0.019 | 0.026 |
| Lat. (◦) | -0.012 | -0.119 | 0.257 | -0.008 | -0.038 | 0.059 |
| Long. (◦) | -0.008 | -0.183 | 0.412 | -0.005 | -0.059 | 0.094 |
| AA (◦) | 0.086 | 0.343 | 0.283 | 0.057 | 0.111 | 0.065 |
| CL (%) | 0.107 | -0.297 | -0.114 | 0.071 | -0.096 | -0.026 |
| SL (%) | -0.183 | 0.1 | 0.272 | -0.121 | 0.032 | 0.062 |
| SN (%) | 0.086 | 0.179 | -0.179 | 0.057 | 0.058 | -0.041 |
| pH (1:5) | -0.1 | -0.022 | -0.021 | -0.066 | -0.007 | -0.005 |
| OM% | 0.06 | -0.088 | -0.52 | 0.04 | -0.028 | -0.119 |
| CaCO_3_ (%) | 0.009 | -0.104 | 0.103 | 0.006 | -0.034 | 0.023 |
| N % | 0.099 | -0.311 | -0.288 | 0.066 | -0.1 | -0.066 |
| P (mg/kg) | -0.151 | -0.14 | 0.114 | -0.1 | -0.045 | 0.026 |
| K (mg/kg) | 0.32 | -0.069 | -0.317 | 0.212 | -0.022 | -0.072 |
| EC (µS/cm) | 0.189 | -0.662 | 0.089 | 0.125 | -0.214 | 0.02 |
| WP (%) | -0.033 | -0.054 | 0.319 | -0.022 | -0.018 | 0.073 |
| FC (%) | -0.306 | -0.084 | 0.008 | -0.202 | -0.027 | 0.002 |
| BD (%) | -0.116 | 0.249 | 0.001 | -0.077 | 0.08 | 0 |
| SPSP (%) | 0.027 | 0.013 | -0.253 | 0.018 | 0.004 | -0.058 |
| AW (%) | 0.07 | -0.072 | -0.194 | 0.046 | -0.023 | -0.044 |
| H | 0.978 | -0.021 | -0.01 | 0.647 | -0.007 | -0.002 |
| S | 0.98 | -0.001 | -0.008 | 0.649 | 0 | -0.002 |
| J | 0.946 | -0.053 | -0.028 | 0.626 | -0.017 | -0.006 |

Note: Variables codes were the same as that of Table 4 and Figure 4
