# Supplementary material for: Spatial distribution of the four invasive plants and their impact on natural communities’ dynamics across the arid and semi-arid environments in northwest Pakistan
Source: Front Plant Sci. 2023 Aug 25;14:1207222. doi: 10.3389/fpls.2023.1207222 (PMC10485558; doi:10.3389/fpls.2023.1207222)
Supplement: Supplementary file 2 [file Table_1.docx]

**Table S1:** Floristic characteristics of the communities in the studied area

| **S. No.** | **Species name** | **Code** | **Family** | **Habit** | **Life form** | **Status** | **Habitat** |
| --- | --- | --- | --- | --- | --- | --- | --- |
| 1 | ***Parthenium hysterophorus* L.** | Ph | Asteraceae | A | H | Invasive | RD |
| 2 | ***Datura innoxia*** | Di | Solanaceae | A | H | Naturalized | RD |
| 3 | ***Xanthium strumarium* L.** | Xs | Asteraceae | A | H | Invasive | RD |
| 4 | ***Silybum marianum* (L.) Geartn** | Sm | Asteraceae | A | H | Invasive | RD |
| 5 | *Acacia nilotica* (L.) Willd. ex Delile | An | Memosaceae | P | T | Native | DS |
| 6 | *Achyranthes aspera* L. | Aa | Solanaceae | A | H | Native | RD |
| 7 | *Achyranthus aspera* L. | Aa | Amaranthaceae | A | H | Native | RD |
| 8 | *Ailanthus* *altissimus* (Mill.) Swingle | Aal | Simaroubaceae | A | T | Invasive | WL |
| 9 | *Ajuga bracteosa* Wall. ex Benth. | Ab | Lamiaceae | A | H | Native | WL |
| 10 | [*Alternanthera pungens* Kunth](https://en.wikipedia.org/wiki/Carl_Sigismund_Kunth) | Ap | Amaranthaceae | A | H | Invasive | RD |
| 11 | *Amaranthus caudatus* L. | Ac | Polygonaceae | A | H | Invasive | AL |
| 12 | *Amaranthus spinosus* L. |  | Amaranthaceae | A | H | Native | AL |
| 13 | *Amaranthus viridis* L. | As | Amaranthaceae | A | H | Invasive | Rd |
| 14 | *Artemisia scoparia* Waldst. & Kit. | As | Asteraceae | P | H | Native | WL |
| 15 | *Asphodelus tenuifolius* Cav. | At | Aspodelaceae | P | S | Invasive | RD |
| 16 | *Avena fatua* L. | Af | Poaceae | A | H | Native | DS |
| 17 | *Avena sativa* L. | As | Poaceae | A | H | Native | RD |
| 18 | *Brassica campestris* L. | Bc | Brassicaceae | A | H | Invasive | AL |
| 19 | *Brossentia papyrifera* (L.) L’Herit. ex Vent | Bp | Moraceae | P | T | Invasive | RD |
| 20 | *Calendula arvensis* L. | Car | Asteraceae | A | H | Native | WL |
| 21 | *Calotropis procera* (Aiton) R. Br. | Cp | Apocyanaceae | A | T | Native | DS |
| 22 | *Cannabis sativa* L. | Cs | Cannabaceae | A | H | Native | WL |
| 23 | *Capsella bursa-pastoris* Moench | Cbp | Asteraceae | A | H | Native | AL |
| 24 | *Carthamus oxycantha* Bieb | Co | Asteraceae | A | H | Invasive | AL |
| 25 | *Cassia occidentalis* L. | Ca | Fabaceae | A | S | Invasive | DS |
| 26 | *Cenchrus ciliaris* L. | Cc | Poaceae | P | H | Native | DS |
| 27 | *Centaurea cyanus* L. | Cc | Asteraceae | A | H | Naturalized | DS |
| 28 | *Chenopodium album* L | Cal | Chenopodiaceae | A | H | Native | RD |
| 29 | *Chrozophora tinctoria* (L.) Raf. | Ct | Euphorbiaceae | A | H | Native | RD |
| 30 | *Cirsium arvense* (L.) Scop. | Ca | Asteraceae | P | H | Native | RD |
| 31 | *Convolvulus arvensis* L. | Ca | Convolvulaceae | P | H | Native | AL |
| 32 | *Cortaderia selloana* (Schult. & Schult.f.) Asch. & Graebn. | Cs | Poaceae | A | H | Native | AL |
| 33 | *Cucurbita pepo* L*.* | Cp | Cucurbitaceae | A | H | Invasive | WL |
| 34 | *Cynodon dactylon* (L.) Pers. | Cd | Poaceae | P | H | Native | AL |
| 35 | *Cyperus rotundus* L. | Cr | Cyperaceae | P | H | Invasive | WL |
| 36 | *Cyprus rotundus* L. | Cr | Cyperaceae | P | H | Invasive | AL |
| 37 | *Desmostachya bipinnata* (L.) Stapf | Db | Poaceae | P | H | Native | AL |
| 38 | *Dichanthium annulatum* (Forssk.) Stapf | Da | Poaceae | P | H | Native | WL |
| 39 | *Dodonaea viscosa* (L.) Jacq. | Dv | Sapindaceae | P | S | Native | AL |
| 40 | *Dysphania ambrosioides* L. | Da | Amaranthaceae | A | H | Native | RD |
| 41 | *Echornia cresipes* (Mart.) Solma in DC | Ec | Pontederiaceae | P | H | Invasive | WL |
| 42 | *Eclipta alba* L. | Ea | Asteraceae | P | H | Invasive | WL |
| 43 | *Erigeron canadensis* (L.) Cronq. | Cc | Asteraceae | A | H | Invasive | WL |
| 44 | *Eryngium caeruleum* Gilib. | Ec | Apiaceae | P | H | Native | AL |
| 45 | *Eucalyptus camaldulensis* Dehnh. | Ec | Brassicaceae | A | H | Invasive | AL |
| 46 | *Euphorbia helioscopia* Dumort. | Eh | Euphorbiaceae | A | H | Native | WL |
| 47 | *Euphorbia hirta* L. | Ehe | Euphorbiaceae | A | H | Invasive | WL |
| 48 | *Euphoria prostrate* Aiton | Ep | Euphorbiaceae | A | H | Invasive | AL |
| 49 | *Ficus carica* L. | Fc | Moraceae | P | T | Invasive | AL |
| 50 | *Fragaria indica* Andrews | Fi | Rosaceae | P | H | Native | WL |
| 51 | *Fumaria indica* Hausskn. | Fi | Papavaraceae | A | S | Naturalized | WL |
| 52 | [*Gymnosporia royleana*Wall. ex M.A. Laws](https://fr.wikipedia.org/w/index.php?title=Gymnosporia_royleana&action=edit&redlink=1) | Gr | [Celastraceae](https://www.google.com/url?sa=t&rct=j&q=&esrc=s&source=web&cd=&ved=2ahUKEwiqw8qXrb_vAhU2SBUIHe9JD1MQs2YoADACegQIBhAM&url=https%3A%2F%2Fen.wikipedia.org%2Fwiki%2FCelastraceae&usg=AOvVaw1Axg00DAb0-Sl9zBhYZP8W) | A | S | Native | WL |
| 53 | *Hackelia virginiana* L. | Hv | Boraginaceae | P | H | Native | DS |
| 54 | *Helianthus annus* L. | Ha | Asteraceae | P | H | Invasive | WL |
| 55 | *Heliotropium curassavicum* L. | Hc | Brassicaceae | P | H | Naturalized | RD |
| 56 | *Hibiscus mutabilis* L. | Hm | Malvaceae | P | S | Invasive | WL |
| 57 | *Hypochaeris radicata* L. | Hr | Asteraceae | A | H | Invasive | DS |
| 58 | *Indigofera gerardiana* Graham | Ig | Fabaceae | P | S | Native | WL |
| 59 | *Justicia adhatoda* L. | Ja | Acanthaceae | P | H | Native | AL |
| 60 | *Lathyrus sativus* L. | Ls | Fabaceae | P | H | Native | AL |
| 61 | *Lipidium sativa* L. | Ls | Brassicaceae | A | H | Invasive | RD |
| 62 | *Mangifera indica* L. | Mi | Anacardiaceae | P | T | Invasive | RD |
| 63 | *Medicago sativa* L. | Ms | Fabaceae | P | H | Invasive | WL |
| 64 | *Melia azedarach* L. | Maz | Meliaceae | P | T | Native | RD |
| 65 | *Melilotus indicus* (L.) All. | Mi | Fabaceae | A | H | Invasive | WL |
| 66 | *Mentha longifolia* (L.) Huds. | Ml | Lamiaceae | P | H | Native | AL |
| 67 | *Mentha spicata* L. | Ms | Lamiaceae | P | H | Invasive | AL |
| 68 | *Mirabilis jalapa* L. | Mj | Nyctaginacea | P | H | Invasive | RD |
| 69 | *Morus alba* L. | Ma | Moraceae | P | T | Native | Al |
| 70 | *Narcissus tazetta* DC. | Nt | Amaryllidaceae | P | H | Native | AL |
| 71 | *Nasturtium officinale*  W.T. Aiton | No | Brassicaceae | P | H | Native | WL |
| 72 | *Origanum vulgare* (Linn) | Ov | Lamiaceae | P | H | Native | AL |
| 73 | *Otostegia lambata* Benth. | Ol | Lamiaceae | A | H | Native | WL |
| 74 | *Oxalis carniculata* L. | Oc | Amaranthaceae | P | H | Native | AL |
| 75 | *Persicaria maculosa* S.F.Gray | Pm | Polygonaceae | A | H | Native | WL |
| 76 | *Phalaris caroliniana* Walter | Pc | Poaceae | A | S | Naturalized | WL |
| 77 | *Phalaris minor* Retz. | Pm | Poaceae | P | H | Invasive | WL |
| 78 | *Phragmites karka* (Retz.) Trin. ex Steud. | Pk | Poaceae | P | S | Native | WL |
| 79 | *Physalis minima* L. | Pm | Solanaceae | A | H | Native | RD |
| 80 | *Pinus roxburghii* Sarg. | Pr | Pinaceae | P | T | Native | AL |
| 81 | *Poa annua* Fr. ex Andersson | Pan | Poaceae | A | H | Invasive | RD |
| 82 | *Populus nigra* L. | Pn | Saliaceae | P | T | Naturalized | AL |
| 83 | *Prosopis julifolia* (Sw.) DC. | Pj | Fabaceae | P | T | Invasive | AL |
| 84 | *Ricinus communis* L. | Rc | Solanaceae | P | S | Invasive | WL |
| 85 | *Robinia pseudoacacia* L. | Rp | Fabaceae | P | T | Invasive | WL |
| 86 | *Rubus fruticosus* L. | Rf | Rosaceae | P | S | Native | DS |
| 87 | *Rumex dentatus* L. | Rd | Myrtaceae | P | T | Invasive | AL |
| 88 | *Rumex hastatus* D.Don | Rh | Polygonaceae | P | H | Native | AL |
| 89 | *Salvia moorcroftiana* Wall. ex Benth. | Sm | Lamiaceae | P | H | Native | AL |
| 90 | *Solanum melongena* (Mill.) Dunal | Sm | Solanaceae | A | H | Invasive | AL |
| 91 | *Solanum nigrum* L. | Sn | Solanaceae | A | H | Invasive | WL |
| 92 | *Solanum xanthocarpum* Schrad. & J.C. Wendl. | Sx | Solanaceae | P | H | Native | WL |
| 93 | *Sonchus asper* L. | So | Oxilidaceae | A | H | Native | AL |
| 94 | *Spergula arvensis* L. | Sa | Caryophyleaceae | A | H | Native | AL |
| 95 | *Stylosanthes humilis* Kunth | Sh | Fabaceae | A | H | Native | AL |
| 96 | *Tagetes erectus* L | Te | Asteraceae | A | H | Invasive | WL |
| 97 | *Tagetes minuta* L. | Tm | Asteraceae | A | H | Invasive | WL |
| 98 | *Taraxicum officinale* Weber | To | Asteraceae | A | H | Naturalized | RD |
| 99 | *Trianthema portulacastrum* L. | Tp | Aizoaceae | A | H | Invasive | RD |
| 100 | *Tribulus terrestris* L. | Tt | Zygophyllaceae | A | H | Invasive | WL |
| 101 | *Trifolium repens* L. | Tr | Fabaceae | A | H | Native | WL |
| 102 | *Utrica dioica* L. | Ud | Urticaceae | P | H | Native | RD |
| 103 | *Verbascum Thapsus* L. | Vt | Scrophulariaceae | B | H | Native | DS |
| 104 | *Zanthoxylum armatum* DC. | Za | Lamiaceae | P | T | Native | RD |
| 105 | *Zea mays* L. | Zma | Poaceae | A | H | Invasive | AL |
| 106 | *Ziziphus nummularia* Aubrév. | Zn | Rhamnaceae | P | S | Native | DS |
| 107 | *Ziziphus oxyphylla* Edgew. | Zo | Rhamnaceae | P | S | Native | DS |

Note: P= perennial herb, D= deciduous tree, A= annual tree, P= perennial, A= annual, E= Evergreen,, B= biennial, V=vine, A=, H = Herbs, S = Shrubs, T= Tree, Al =Arable lands, W=Waste places, G=Grasslands, F=Forests, M=Marshes, WC=Water courses, D=Drier slopes, S=Sandy stream/riversides), MS=Moist Shady places, R=Rock crevices, GY=Graveyards, , Rd = Road, Fl=Felds
